# A Silicon Nanomembrane Detector for Matrix-Assisted Laser Desorption/Ionization Time-of-Flight Mass Spectrometry (MALDI-TOF MS) of Large Proteins

**DOI:** 10.3390/s131013708

**Published:** 2013-10-11

**Authors:** Jonghoo Park, Robert H. Blick

**Affiliations:** 1 Department of Electrical Engineering, Kyungpook National University, 80 Daehak-ro, Buk-gu, Daegu 702-701, Korea; E-Mail: jonghoopark@knu.ac.kr; 2 Department of Electrical and Computer Engineering, University of Wisconsin-Madison, Madison, WI 53706, USA; 3 Center for Hybrid Nanostructures (CHYN), Institute of Applied Physics, University of Hamburg, Jungiussstr. 11, Hamburg 20355, Germany

**Keywords:** silicon nanomembrane, MALDI TOF, mass spectrometry, phonon, ion detector, NEMS

## Abstract

We describe a MALDI-TOF ion detector based on freestanding silicon nanomembrane technology. The detector is tested in a commercial MALDI-TOF mass spectrometer with equimolar mixtures of proteins. The operating principle of the nanomembrane detector is based on phonon-assisted field emission from these silicon nanomembranes, in which impinging ion packets excite electrons in the nanomembrane to higher energy states. Thereby the electrons can overcome the vacuum barrier and escape from the surface of the nanomembrane via field emission. Ion detection is demonstrated of apomyoglobin (16,952 Da), aldolase (39,212 Da), bovine serum albumin (66,430 Da), and their equimolar mixtures. In addition to the three intact ions, a large number of fragment ions are also revealed by the silicon nanomembrane detector, which are not observable with conventional detectors.

## 1. Introduction

In time-of-flight (TOF) mass spectrometry (MS) [1], large molecules—the proteins—are desorbed and ionized by the matrix-assisted laser desorption/ionization (MALDI) [2,3] technique before being accelerated in an electric field, and directed into the flight tube where they are separated by their mass-to-charge (*m*/*z*) ratio. Thereby the ions of different masses reach the detector at different times and velocities, while the kinetic energy of the ions remains the same, regardless of their mass. The operating principle of the ion detectors used in most TOF mass spectrometers, e.g., the microchannel plates (MCP) and dynodes, are based on secondary electron emission [4]. In these detectors, the incident ion generates secondary electrons, which are then multiplied via a sequential cascade process. The secondary electron emission yield decreases as the velocity of the incident ions decreases [5]. This leads to a remarkable decrease in detection sensitivity for larger ions, which drift more slowly down to the detector than lighter ones. Therefore, MALDI-TOF remains predominantly restricted to the mass analysis of biomolecules with a mass below about 300,000 Daltons [6].

Recently, we demonstrated MALDI-TOF MS of proteins using nanomembranes as ion detectors. Nanomembranes are extremely versatile structures with thicknesses in the range of a few 10 nm to several 100 nm, while the lateral dimensions can be orders of magnitude larger than the thickness. The main attraction of nanomembranes as ion detectors lie in their unique mechanical, *i.e.*, they can be excited thermomechanically [7,8], and thermal properties, that is in particular the membranes are thinner than the phonon mean free path [9]. *The nanomembrane detector utilizes the kinetic energy of the accelerated ion, which is the same regardless of its mass*. Therefore the sensitivity of the nanomembrane detectors is largely independent of ion mass. The operating principles of these nanomembrane detectors are based on: (*i*) quasi-dynamic mode of vibration in Al/Si_3_N_4_/Al nanomembranes [7,8] and (*ii*) phonon-assisted filed emission (PAFE) in silicon nanomembranes [9].

In the quasi-dynamic mode the operating principle of the detectors is based on the transduction of the kinetic energy of the ions into mechanical oscillations of the nanomembrane. When accelerated ion packets bombard the Al/Si_3_N_4_/Al nanomembrane, the kinetic energy is transformed into thermal energy. This raises the temperature in the vicinity of the impact site and causes a non-uniform temperature distribution across the nanomembrane. The temperature gradient leads to thermomechanical forces, which excite mechanical oscillations of the nanomembrane. These oscillations alter the distance between the extraction gate and the nanomembrane surface, which in turn modulates the electric field intensity between them. This causes an oscillating Fowler-Nordheim field emission current [10]. The deformation and vibrations of the nanomembrane due to the thermal force and the applied DC voltage onto the nanomembrane can be expressed by Equation (1) [11]:

(1) $$\rho h\ddot{\zeta }+D\Delta^{2}\zeta -F\Delta \zeta =\frac{-E\alpha }{3(1-2\sigma )}\nabla T-\frac{1}{2}V^{2}\frac{dC}{d\zeta }$$

where *ζ* is the vertical displacement, *ρ* is the density, *h* is the thickness of the nanomembrane, *D* = *Eh*^3^/12(1 − *σ*^2^) is the flexure rigidity, *E* is Young's modulus, *σ* is the Poisson ratio, *F* is the stretching force per unit length of the edge of the nanomembrane, *α* is the thermal expansion coefficient, *T* is the temperature increase above a uniform ambient level, *V* is the extraction gate voltage, and *C* is the capacitance between the nanomembrane and the extraction gate.

The characteristic frequencies for the vibration of the nanomembrane can be expressed by:

(2) $$f_{i,j}=\frac{1}{2l}\sqrt{\frac{F(i^{2}+j^{2})}{\rho h}}$$

where *l* is the side length of the nanomembrane, *i* and *j* are integers.

Ion detection using this approach was demonstrated over a broad range with angiotensin (1,296 Da), equimolar mixture of insulin (5,729 Da), bovine serum albumin (BSA, 66,429 Da), and immunoglobulin G (IgG, 150,000 Da). The detectors demonstrated that the sensitivity was not highly dependent on the mass and the upper mass limit of the detector was estimated to be 1.5 MDa.

In the second case phonon-assisted field emission from silicon nanomembranes is employed: the detector converts thermal energy deposited by the ion bombardment directly to an electrical signal. The temperature rise in the vicinity of the impact site produces non-equilibrium phonons, which carry thermal energy away from the impact site. These non-equilibrium phonons deliver sufficient thermal energy to the electrons within the nanomembrane and excite them to higher energy states, thereby allowing them to overcome the vacuum barrier (∼4.6 eV for undoped silicon), which is lowered and thinned by external electrical fields, and escape from the nanomembrane surface via field emission. This results in the increased field emission current and can be referred to as phonon-assisted field emission and is governed by Equation (3) [12]:

(3) $$J(T,F)=\frac{mek_{B}}{2\pi \hbar^{3}}T^{2}e^{-(W-\Delta W)/k_{B}T}$$

where *W* is the work function of the material, and Δ*W* = (*eF*/4*πε*_0_)^1/2^ is the field-dependent correction to the work function due to tunneling through the barrier with *F* being the electric field strength, *T* is the temperature, and *e* and *m* are the electron's charge and mass, respectively.

When electrons escape from the nanomembrane by PAFE, they carry an energy amount equal to the average energy difference between the emitted and replaced electrons. This results in cooling of the cathode-side (front-side) of the nanomembrane [13–15]. The non-equilibrium phonon-assisted field emission from the nanomembrane coupled to an effective cooling of the nanomembrane via field emission allows mass analysis of megadalton ions with high mass resolution at room temperature. Ion detection using this approach was demonstrated in MALDI-TOF analysis of IgG and immunoglobulin M (IgM, ∼1 MDa) [9].

## 2. Experiments

In addition to detecting high molecular weight proteins with high mass resolution, mass analysis of complex protein mixtures such that derived from tissue samples or biological fluids is particularly important, Because it can potentially be used to discriminate disease-specific proteomic patterns, which provide diagnostic tools for clinical applications. We demonstrate here mass analysis using phonon-assisted field emission from silicon nanomembranes for apomyoglobin (16,952 Da), aldolase (39,212 Da), bovine serum albumin (66,430 Da), and an equimolar mixture of these three proteins.

### 2.1. Fabrication of Silicon Nanomembranes

For preparing the nanomembrae the 600 nm thick silicon layer of the silicon-on-insulator (SOI) is thinned down to 180 nm by thermal oxidation. A 150 nm thick layer of silicon nitride is deposited on both sides of the wafer using low-pressure chemical vapor deposition (LPCVD), forming a layer of Si_3_N_4_/SiO_2_/SOI/SiO_2_/Si_3_N_4_. Two square membranes (2 × 2 mm^2^ each) are defined by optical lithography and etched through via reactive ion etching (RIE) of Si_3_N_4_ on the backside of the wafer. The SiO_2_ layer on the backside of the wafer is removed by 6:1 buffered oxide etch (BOE). The Si_3_N_4_/SiO_2_/Si/SiO_2_ membranes are formed by anisotropic etching of the silicon substrate using potassium hydroxide (KOH) solution. Finally, silicon nanomembranes are formed by RIE of top Si_3_N_4_ layer followed by 6:1 BOE to etch the SiO_2_ on both sides of the silicon layer.

### 2.2. Configuration of the Detector

**Figure 1.**
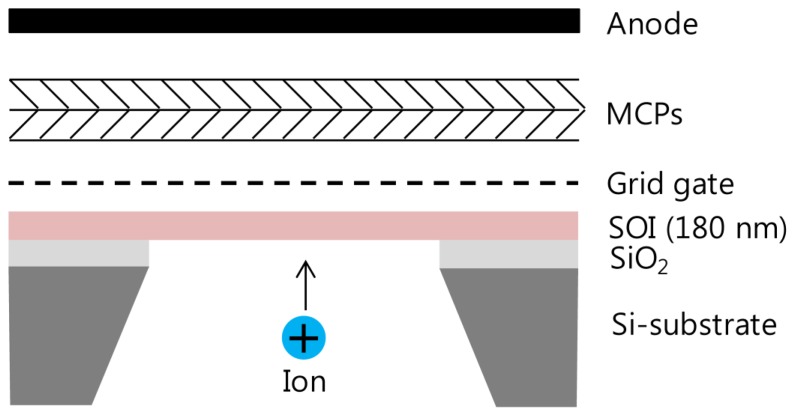
Schematic of the detector configuration, consisting of a 180 nm thick silicon nanomembrane, an extraction gate, MCP, and an anode.

The silicon nanomembrane detector consists of four parts, a silicon nanomembrane, an extraction gate, microchannel plates (MCP)s, and an anode, as shown in Figure 1. The number of electrons emitted by PAFE is amplified by MCPs in a Chevron configuration and collected by an anode. The signal collected by the anode is recorded by an oscilloscope in real time after low pass filtering. The silicon nanomembrane detector is placed at the end of the flight tube of a commercial MALDI-TOF mass spectrometer (Voyager-DE STR, Perseptive Biosystems, Framingham, MA, USA).

### 2.3. Protein Sample Preparation

A 10 pmol/μL concentration of apomyoglobin (16,952 Da), aldoase (39,212 Da), and bovine serum albumin (66,430 Da) are dissolved in 0.1% trifluoroacetic acid (TFA) solution. A 10 mg/mL concentration of 3,5-dimethoxy-4-hydroxycinnamic acid (sinapinic acid) matrix was dissolved in the 50% acetonitrile in 0.05% TFA solution. A 10 pmol/μL concentration of apomyoglobin, aldose, and bovine serum albumin are mixed for the equimolar mixture (3.3 pmol/μL each) sample. All proteins, matrix, and solvents are purchased from Sigma-Aldrich (St. Louis, MO, USA).

## 3. Results

**Figure 2.**
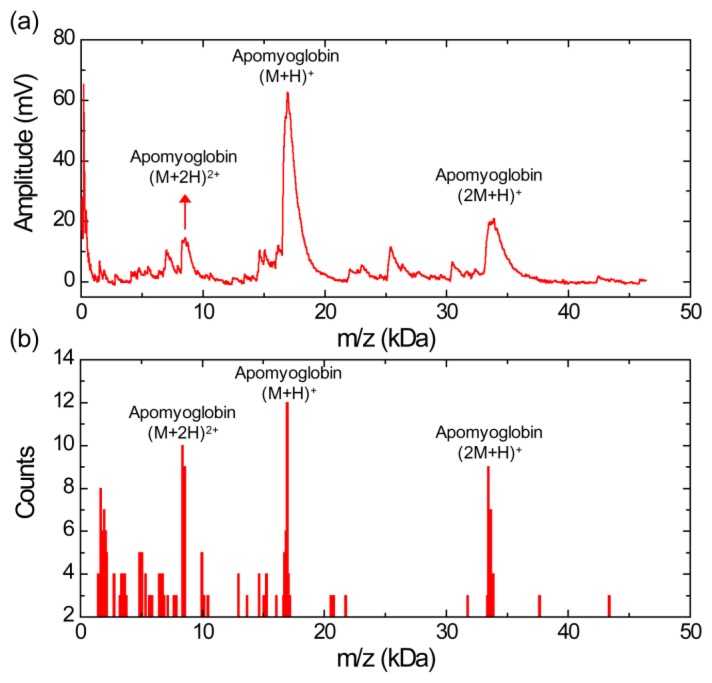
(**a**) MALDI-TOF mass spectrum for apomyoglobin with sinapinic acid matrix obtained using silicon nanomembrane detector, showing singly charged ion, doubly charged ion, and singly charged dimer ion. (**b**) Histogram of the MALDI-TOF mass spectrum, showing a relative abundance of apomyoglobin ions in the sample under test.

Figure 2a shows a MALDI mass spectrum obtained using the silicon nanomembrane detector for apomyoglobin with sinapinic acid matrix. In addition to the three major peaks, corresponding to the singly charged apomyoglobin (*m/z* of 16,948 Da), doubly charged ions (*m/z* of 8,556 Da) and singly charged dimer ions (*m/z* of 33,876 Da), multiple peaks in masses lower than mass of the apomyoglobin are also observed with much lower amplitude. These small peaks are frequently shown in our experiments. We speculate that these peaks are fragment ions, which are produced by high laser power. The laser intensity for the spectrum is set to be slightly higher than the threshold laser intensity for BSA in an equimolar mixture of apomyoglobin, aldolase, and BSA, which will be discussed below. The mass spectrum is obtained by averaging 10 laser shots. The acceleration voltage, the voltage between the silicon nanomembrane and the extraction gate set to 23.5 kV and 1.4 kV, respectively. These instrument conditions are used for all experiments in this article.

Figure 2b shows a histogram of the MALDI-TOF mass spectrum for apomyoglobin. The peak values were extracted from 30 different MALDI-TOF mass spectra gathered for the histogram and each spectrum was obtained by averaging 10 laser shots. It shows a relative abundance of apomyoglobin ions in the sample under test with a bin size of 100 Da. The *m/z* values of the peaks for singly charged apomyoglobin ions are distributed in *m/z* range from 16,650 to 17,150 Da (center value of the bin). The *m/z* value of the most abundant fragment ions are distributed in 1,650 and 1,950 Da (center value of the bin).

**Figure 3.**
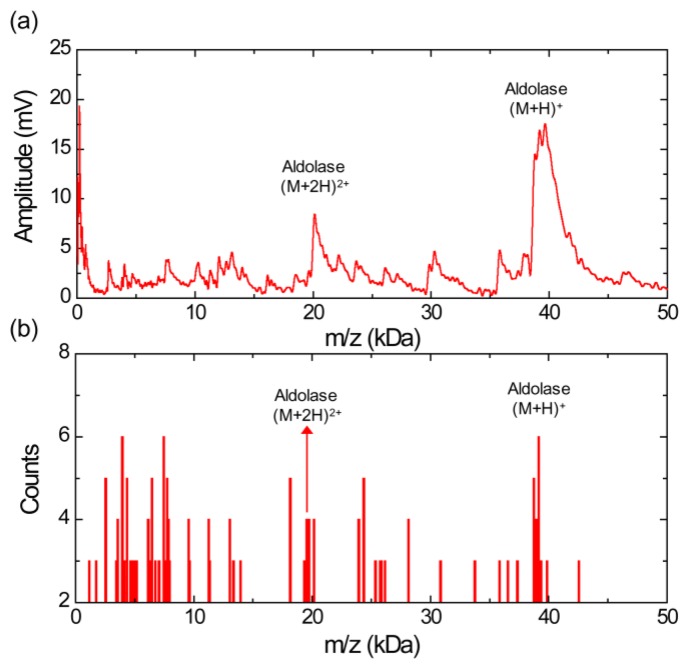
(**a**) MALDI-TOF mass spectrum for aldolase with siniapinic acid matrix obtained using the silicon nanomembrane detector, showing singly and doubly charged aldolase ions, and a large number of fragment ions. (**b**) Histogram of the MALDI-TOF mass spectrum, showing a relative abundance of aldolase ions in the sample under test.

Figure 3a shows a MALDI mass spectrum obtained using the silicon nanomembrane detector for aldolase with sinapinic acid matrix. It clearly shows singly (*m/z* of 39,192 Da) and doubly charged aldolase ions (*m/z* of 20,122 Da) as well as fragments ions over this mass range. The histogram of the MALDI-TOF mass spectrum, Figure 3b, shows the relative abundance of the fragment ions with respect to the singly charged aldolase ions with a bin size of 100 Da. The *m/z* values of the peaks for the singly charged aldolase ions are distributed in *m/z* range from 38,950 to 39,350 Da (center value of the bin). Similar to the Figure 2, a large number of fragment ions are shown and the *m/z* values of five most abundant fragment ions are distributed in the bin of 2,550, 3,950, 4,350, 6,450, 7,450, 7,750, 18,150 and 24,350 Da (center of the bin).

**Figure 4.**
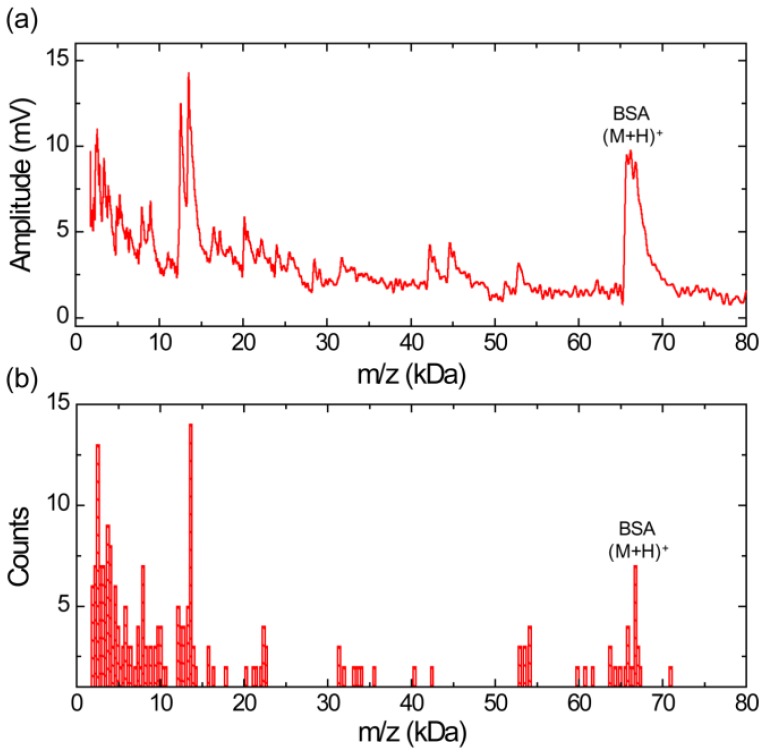
(**a**) MALDI-TOF mass spectrum for BSA with a sinapinic acid matrix obtained using silicon nanomembrane detector, clearly showing singly charged ions. (**b**) Histogram of the MALDI-TOF mass spectrum, showing a relative abundance of apomyoglobin ions in the sample under test.

Figure 4a shows a MALDI TOF mass spectrum obtained using silicon nanomembrane for BSA with sinapinic acid matrix. The spectrum clearly shows singly charged BSA ions at *m/z* of 66,468 Da and large fragment ion peak at *m/z* of 13,493 Da. The fragment ion peak at *m/z* of 13,493 is frequently shown and is the most abundant ion in the sample under test as shown in Figure 4b, represented in histogram. The bin size of the histogram is 300 Da. The histogram shows a large number of fragment ions in the mass range below 15 kDa.

**Figure 5.**
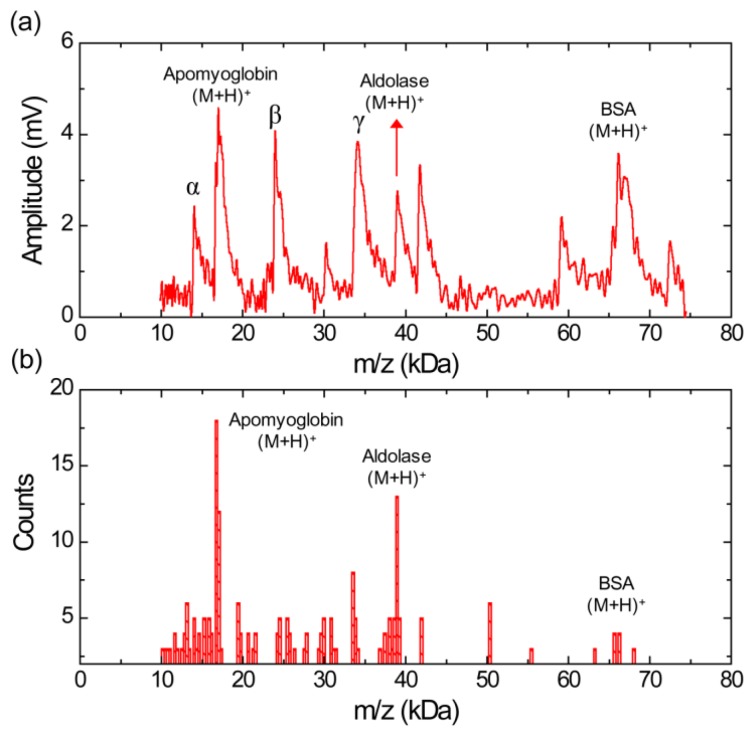
(**a**) MALDI-TOF mass spectrum for an equimolar mixture of apomyoglobin, aldolase, and BSA with a sinapinic acid matrix obtained using silicon nanomembrane detector. (**b**) Histogram of the MALDI-TOF mass spectrum, showing a relative abundance of each protein in equimolar mixtures.

Figure 5a shows a MALDI-TOF mass spectrum using silicon nanomembrane for an equimolar protein mixture (3.3 pmol/μL each) of aldolse, apomyoglobin, and BSA in sinapinic acid matrix. It clearly shows singly charged apomyoglobin, aldolase, and BSA ions at *m/z* of 17,006, 39,007, and 66,165 Da, respectively. The peak at *m/z* of 14,045 Da (labeled as α) was frequently shown in the mass spectrum for BSA (Figure 4a) and the histogram for BSA (Figure 4b). The peaks at *m/z* of 23,991 Da (labeled as β) are also shown in the histogram (Figure 3b) for aldolase. The peak at *m/z* of 34,128 Da (labeled as γ) is dimer for apomyoglobin as shown in Figure 2. Figure 5b shows a histogram of the equimolar protein mixture with a bin size of 300 Da. Three most abundant ions are singly charged apomyoglobin, aldolase and doubly charged BSA with center of bin value of 16,750, 38,950, and 33,550 Da. The counts for the singly charge BSA is much smaller than that of apomyoglobin and aldolase, indicating that BSA is desorbed less preferentially than other two proteins.

## 4. Conclusions

We have demonstrated the feasibility of MALDI-TOF mass analysis over a broad mass range of proteins and complex protein mixtures using the silicon nanomembrane detector. The detector reveals not only the intact proteins, but also a large number of fragment ions in the complex protein mixtures. Our study suggests that this operating principle of ion detection offers a huge potential for in-source-decay [16,17] analysis in MALDI-TOF mass spectrometry for large ions and complex protein mixtures. The sensitivity of the detector can be further improved by increasing the total size of the silicon nanomembrane, as the size of silicon nanomembranes in this work is about 200 times smaller than that of a typical microchannel plate (MCP) detector with 2″ diameter. The scaling of the silicon nanomembranes size can be easily achieved by CMOS-compatible fabrication processes.

## References

[1] Wiley W.C., Mclaren I.H. (1955). Time-of-flight mass spectrometer with improved resolution. Rev. Sci. Instrum..

[2] Tanaka K., Waki H., Ido Y., Akita S., Yoshida Y., Yoshida T., Matsuo T. (1988). Protein and polymer analyses up to m/z 100,000 by laser ionization time-of-flight mass spectrometry. Rapid Commun. Mass Spectrom.

[3] Karas M., Hillenkamp F. (1988). Laser desorption ionization of proteins with molecular masses exceeding 10,000 daltons. Anal. Chem..

[4] Geno P.W. (1992). Mass Spectrometry in the Biological Sciences: A Tutorial, Ion Detection in MS.

[5] Chen X., Westphall M.S., Smith L.M. (2003). Mass spectrometric analysis of DNA mixtures: Instrumental effects responsible for decreased sensitivity with increasing mass. Anal. Chem..

[6] De Hoffmann E., Stroobant V. (2007). Mass Spectrometry: Principles and Applications.

[7] Park J., Qin H., Scalf M., Hilger R.T., Westphall M.S., Smith L.M., Blick R.H. (2011). A mechanical nanomembrane detector for time-of-flight mass spectrometry. Nano Lett..

[8] Park J., Kim H., Blick R.H. (2012). Quasi-dynamic mode of nanomembranes for time-of-flight mass spectrometry of proteins. Nanoscale.

[9] Park J., Aksamija Z., Shin H.-C., Kim H., Blick R.H. (2013). Phonon-assisted field emission in silicon nanomembranes for time-of-flight mass spectrometry of proteins. Nano Lett..

[10] Fowler R.H., Nordheim L. (1928). Electron emission in intense electric fields. Proc. R. Soc. Lond. Ser. A..

[11] Landau L.D., Lifshitz E.M. (1986). Theory of Elasticity.

[12] Murphy E.L., Good H.R. (1956). Thermionic emission, field emission, and the transition region. Phys. Rev..

[13] Richardson O.A. (1903). The electrical conductivity imparted to a vacuum by hot conductors. Philos. Trans. R. Soc. Lond. Ser. A..

[14] Nottingham W.B. (1941). Remarks on energy losses attending thermionic emission of electrons from metals. Phys. Rev..

[15] Charbonnier F.M., Strayer R.W., Swanson L.W., Martin E.E. (1964). Nottingham effect in field and T-F emission: Heating and cooling domains, and inversion temperature. Phys. Rev. Lett..

[16] Brown R.S., Lennon J.J. (1995). Sequence-specific fragmentation of matrix-assisted laser-desorbed protein/peptide ions. Anal. Chem..

[17] Katta V., Chow D., Rohde M. (1998). Applications of in-source fragmentation of protein ions for direct sequence analysis by delayed extraction MALDI-TOF mass spectrometry. Anal. Chem..

